# Prevalence of depression, anxiety, and stress among first responders for medical emergencies during COVID-19 pandemic: A meta-analysis

**DOI:** 10.7189/jogh.12.05028

**Published:** 2022-07-25

**Authors:** Garry Huang, Hsin Chu, Ruey Chen, Doresses Liu, Kondwani Joseph Banda, Anthony Paul O’Brien, Hsiu-Ju Jen, Kai-Jo Chiang, Jeng-Fong Chiou, Kuei-Ru Chou

**Affiliations:** 1 School of Health Care Administration, College of Management, Taipei Medical University, Taipei, Taiwan; 2 Australasian College of Paramedicine, Australia; 3 Australian Institute of Project Management, Australia; 4 Institute of Aerospace and Undersea Medicine, School of Medicine, National Defense Medical Center, Taipei, Taiwan; 5 Department of Neurology, Tri-Service General Hospital, National Defense Medical Center, Taipei, Taiwan; 6 Department of Nursing, Taipei Medical University-Shuang Ho Hospital, New Taipei, Taiwan; 7 Post-Baccalaureate Program in Nursing, College of Nursing, Taipei Medical University, Taipei, Taiwan; 8 School of Nursing, College of Nursing, Taipei Medical University, Taipei, Taiwan; 9 Department of Nursing, Wan Fang Hospital, Taipei Medical University; 10 Center for Nursing and Healthcare Research in Clinical Practice Application, Wan Fang Hospital, Taipei Medical University, Taipei, Taiwan; 11 Endoscopy Unit, Surgery Department, Kamuzu Central Hospital, Lilongwe, Malawi; 12 Clinical Nursing, Centre for Practice Opportunity and Development, Australia; 13 School of Nursing and Midwifery, Peninsula campus, Monash University, Frankston, Victoria, Australia; 14 Department of Nursing, Tri-Service General Hospital Taipei, Taiwan; 15 School of Nursing, National Defense Medical Center, Taipei, Taiwan; 16 Department of Radiology, School of Medicine, College of Medicine, Taipei Medical University, Taipei, Taiwan; 17 Department of Radiation Oncology, Taipei Medical University Hospital, Taipei, Taiwan; 18 Psychiatric Research Center, Taipei Medical University Hospital, Taipei, Taiwan; 19 Neuroscience Research Center, Taipei Medical University, Taipei, Taiwan

## Abstract

**Background:**

The COVID-19 pandemic has been shown to cause enormous psychological burden among health care workers, including first responders. However, psychological well-being of first responders, essential in the fight against COVID-19 pandemic, has often been ignored. We performed the first meta-analysis to explore the prevalence of 1) depression, 2) anxiety, and 3) stress among first responders for medical emergencies during the COVID-19 pandemic.

**Methods:**

A comprehensive search was conducted in Embase, CINAHL, Web of Science, PsychInfo, PubMed, and the WHO COVID-19 database from 2020. The Freeman-Tukey double-arcsine transformation model in R-software determined the pooled prevalence and Comprehensive Meta-Analysis for associated factors of depression, anxiety, and stress with corresponding 95% confidence intervals (CI). The Cochrane Q, τ^2^, and *I^2^* statistics were used to examine heterogeneity. Sub-group analysis was conducted to identify moderator variables.

**Results:**

We identified 765 records, from which 17 studies were included with 8096 first responders. The pooled prevalence was 31% (95% CI = 21%-41%) for depression; 67% (95% CI = 64%-70%) for mild depression, 24% (95% CI = 17%-31%) for moderate depression, and 16% (95% CI = 4%-34%) for severe depression. The pooled prevalence for anxiety was 32% (95% CI = 20%-44%); 60% (95% CI = 46%-73%) for mild anxiety, 27% (95% CI = 14%–42%) for moderate anxiety, and 14% (95% CI = 7%-22%) for severe anxiety. The pooled prevalence for stress was 17% (95% CI = 4%-34%); 58% (95% CI = 38%-77%) for mild stress, 22% (95% CI = 5%-44%) for moderate stress, and 19% (95% CI = 5%-37%) for severe stress. The prevalence of depression was 37% (95% CI = 25%-52%) for paramedics, 28% (95% CI = 12%-54%) for EMS personnel and 22% (95% CI = 13%-33%) for police. Similarly, the prevalence of anxiety was 38% (95% CI = 20%-60%) for paramedics, 28% (95% CI = 11%-53%) for EMS personnel, and 19% (95% CI = 10%-32%) for police. Married responders were likely at risk for depression (1.50, 95% CI = 1.26-1.78) and anxiety (1.94, 95% CI = 1.62-2.33), while unmarried responders were less likely at risk for depression (0.67, 95% CI = 0.56-0.79) and anxiety (0.50, 95% CI = 0.43-0.63).

**Conclusions:**

High prevalence of depression, anxiety, and stress during the COVID-19 pandemic among first responders for medical emergencies emphasizes the need for monitoring their psychological well-being. Early assessment and management of mild depression, anxiety, and stress among first responders are crucial in preventing progression into moderate and severe types.

The COVID-19 pandemic has been a major public health concern globally, causing significant physical, physiological, and psychological negative health outcomes in all countries [1,2]. First responders, including paramedics, firefighters, ambulance personnel, emergency medical technicians (EMTs), emergency medical service (EMS) personnel, and police, play a vital role in providing and coordinating essential community first response for pre-emergency or out-of-hospital medical services [3,4]. First responders often face direct and indirect distress and traumatic events due to the uncertain nature of their workplaces, causing significant physical, physiological, and psychological health burdens including depression, anxiety, and stress [1,3,4]. Moreover, higher rates of negative physiological and psychological burden have been previously observed and reported among first responders compared to the general population [3,5].

Previous research findings have demonstrated increased levels of depression, insomnia, anxiety, chronic fatigue, and stress among health care workers during previous epidemics of severe acute respiratory syndrome (SARS) and Middle East respiratory syndrome (MERS) [6-8]. Despite reports of high prevalence of psychological burden pre-COVID-19 pandemic, these front-line responders have been at the epicentre of the global fight against the COVID-19 pandemic with limited resources at their disposal. Moreover, their mental health and personal well-being have often been ignored and overlooked by their respective agencies. Furthermore, individual and work-related factors have been shown to contribute to the development and worsening of negative psychological outcomes among first responders [6]. The individual-related factors include age, gender, marital status, education, smoking status, alcohol status, and coping mechanism [6]. Work-related factors include years of work experience, prior training, type of work, profession, peer support, communication, lack of rest, near-death experience, the severity of causalities, previous exposure to disaster, contact with corpses, and awareness of support measures [6]. The current pooled prevalence estimates based on a study by Pertie et al. [9] conducted pre-COVID-19 pandemic are 27% for psychological distress, 15% for depression and anxiety, and 11% for PTSD among ambulance personnel. However, current research shows limited evidence and knowledge on the prevalence of depression, anxiety, and stress during the COVID-19 pandemic among first responders. The previous meta-analysis [9] focused on ambulance personnel only, and first responders continue to be an understudied population among health care workers. Current research findings reveal a gap in the estimation of depression, anxiety, and stress among first responders for medical emergencies during the COVID-19 pandemic. We aimed to explore the pooled prevalence of depression, anxiety, and stress among first responders for medical emergencies using a meta-analysis during the COVID-19 pandemic.

## METHODS

The study protocol was registered with PROSPERO: CRD42022301213 and the reporting of the meta-analysis adhered to the Meta-Analysis of Observational Studies in Epidemiology (MOOSE) and PRISMA statement updated 2020 guidelines [10,11]. A comprehensive search from 2020 was conducted in PsychInfo, PubMed, Embase, Web of Science, CINAHL, WHO COVID-19 database, and reference lists of relevant observational studies, systematic reviews, and meta-analyses. The following keywords were used in combination: (prevalence OR incidence OR epidemiology OR rate OR rates OR number OR proportion OR probability OR event) AND (depression OR anxiety OR stress OR psychological distress) AND (Emergency Medical Services Personnel OR EMS personnel OR ambulance personnel OR fire fighters OR police OR first responders OR paramedics OR emergency medical technicians OR EMTs) AND (COVID-19 or covid-19 OR Corona virus OR SARS-COV 2). A detailed search strategy can be found in Table S1 in the **Online Supplementary Document**. Original authors were contacted to provide data missing from the published studies.

### Study selection

The study inclusion criteria followed the PICOS framework: 1) population: first responders [1,3,4], 2) exposure of interest: depression, anxiety, and stress; 3) comparison: no depression, anxiety, and stress [1,3,4], 4) outcome of interest: incidence, prevalence, or epidemiology 5) study design: observational studies including cross-sectional and prospective cohort studies, and 6) studies with a validated assessment tool. The exclusion criteria were as follows: 1) duplicate studies, 2) non-relevant population studies, 3) randomized controlled trials (RCTs), 4) study protocols, 5) systematic review or meta-analysis studies, 6) studies unrelated to the topic, and 7) studies using non-validated assessment tools. No language restrictions were set.

### Data extraction and study outcomes

GH and KJB independently performed the data extraction from the included studies using standard data extraction forms with the following categories: author, year of publication, age, country, sample size, gender, occupation, type of first responders, study design, assessment method, and psychological outcomes (depression, anxiety, and stress).

The primary outcome was pooled prevalence of depression, anxiety, and stress among first responders for medical emergencies using validated assessment tools. The secondary outcomes were associated factors including 1) being a first responder, including paramedics and EMS, 2) gender (male and female), and 3) marital status (married and unmarried).

### Quality assessment of included studies

The quality of the included studies was examined using Hoy’s Risk of Bias assessment tool for prevalence studies [12]. The tool has 10 domains that assess the internal and external validity of prevalence in observational studies. The external validity of the study domains is assessed in items 1-4, which include selection and non-response bias. The internal validity of the study domains is assessed in items 5-10, which include measurement bias (items 5-9) and analysis-related bias (item 10). Each item is rated as 1 for low risk and 0 for high risk, while quality ranking is determined as follows: 9-10 for low risk of bias study, 7-8 for moderate risk of bias study, and 0-6 for a high of bias risk study (Table S2 in the **Online Supplementary Document**). A third expert reviewer was invited to resolve and address any discrepancies between GH and KJB through discussions.

### Statistical methods

The Freeman-Tukey double-arcsine transformation model was used to estimate the pooled prevalence rate of depression, anxiety, and stress among first responders for medical emergencies during the COVID-19 pandemic with the metaprop package in R software [13,14]. The model uses p_i_ as the proportion estimate from study *i* in the analysis (i = 1,…, N). The pooled prevalence estimate of depression, anxiety, and stress p_i_ was calculated as pi = e_i_/n_i_, with e_i_ being the number of participants with depression, anxiety, and stress, and n_i_ being the total sample size of first responders in the included studies. The model also calculates weighted pooled estimates and performs back-transformation on the pooled estimates to stabilize the within-study variance by using a binomial distribution. Publication bias was examined by Egger’s Regression method and visual inspection of the funnel plots [15].

The DerSimonian-Lard random and fixed-effects model in Comprehensive Meta-Analysis (CMA), Version 2.0 software (Biostat, Englewood, New Jersey, USA) [16] was used to determine the pooled estimates for associated factors 1) being a first responder, 2) gender (male and female), and 3) marital status (married and unmarried). The model uses the inverse variance-weighted mean of the logarithm of OR (odds ratio) with a 95% confidence interval (CI) to estimate the pooled OR for associated factors.

Heterogeneity depression assessment and anxiety prevalence estimates were done to account for variations regarding individual and methodological factors among the included studies. Statistical heterogeneity was examined using an *X^2^*-based test using Cochran's Q statistic (*P* = 0.10), τ^2^ statistic, and the *I^2^* statistic quantified heterogeneity with a score of 25% as low, 50% as moderate, and 75% as high [17].

### Moderator analysis

Moderator analysis was performed among the included studies using pre-specified individual and methodological factors to account for identified statistical heterogeneity [18]. Sub-group analysis was performed for categorical variables, including 1) type of first responders (paramedics, EMS personnel, and police), 2) continent (Africa, Asia, Europe, North America, and South America), 3) type of interview (structured and unstructured), 4) assessment tools, 5) study design (cross-sectional and prospective cohort), 6) study type (web-based and face-to-face), 7) country status (high income, middle income, and low income), 8) sample size (<200 and ≥200), 9) setting (urban & rural and urban), 10) study quality (high and moderate), 11) number of cases and mortality rate for COVID-19 (top 5 countries and non-top 5 countries).

### Ethical approval

No ethical approval was required for the current meta-analysis, as it used secondary data from previously published studies in which informed consent was sought from the participants.

## RESULTS

### Study characteristics

**Figure 1:**
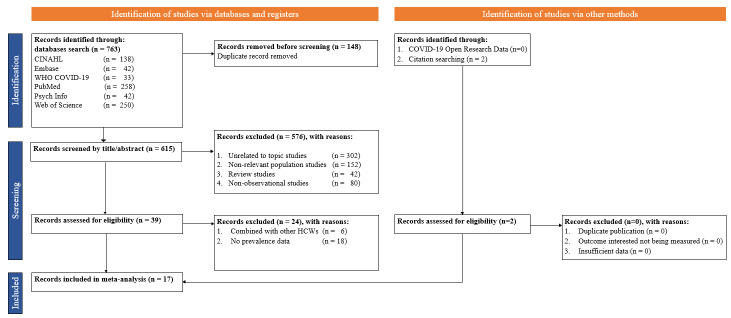
Flowchart for study selection.

We identified 765 studies from PubMed, Web of Science, CINAHL, Embase, WHO COVID-19 database, and PsychInfo from which a total of 17 studies [19-35] published between 2020 and 2022 were included in the current meta-analysis (**Figure 1**). The studies comprised a total of 8096 first responders, including paramedics, police, and EMS. Five studies were done in North and South America, nine in Asia, two in Europe, and one in Africa. Regarding study quality, seven studies had low risk of bias, while ten studies had moderate risk of bias. 16 studies were conducted using a cross-sectional study design, while one study used a prospective cohort design. Of these studies, 13 studies were web-based surveys while four studies were face-to-face surveys (**Table 1**).

**Table 1:** Study characteristics

Author (year), country	Mean age (years), sample (n), gender (M/F/NA)	Study design	Type of first responders	Occupation	Psychological outcome (tool)	Prevalence, (n and %)	Study Quality (score – rating)
Alah et al, (2021), Qatar [19]	NA, n = 37 (NA)	Cross-sectional, Web-based	Paramedics	Professional	Depression (PHQ-9), Anxiety (GAD-7), Stress (IES**-**R)	7 (23.3), 10 (33.3), 10 (33.3)	7 – Moderate Risk
Apaza-Llantoy et al, (2021), Peru [20]	NA, n = 210 (NA)	Cross-sectional, Self-administered	Police	Professional	Depression (DASS-21), Anxiety (DASS-21), Stress (DASS-21)	24 (11.4), 21 (10.0), 16 (7.6)	9 – Low Risk
Dreher et al, (2021), Germany [21]	32.0, n = 1537 (1278/257)	Cross-sectional, Web-based	EMS personnel	Professional	Depression (PHQ-2), Anxiety (GAD-2)	235 (15.3), 247 (16.1)	9 – Low Risk
Grover et al, (2020), India, [22]	36.9, n = 124 (79/45)	Cross-sectional, Web-based	Police	Professional	Depression (PHQ-4), Anxiety (PHQ-4)	112 (18.0), 66 (10.6)	9 – Low Risk
Gupta et al, (2020), India [23]	NA, n = 135 (NA)	Cross-sectional, Web-based	Paramedics	Professional	Depression (HADS), Anxiety (HADS)	51 (14.4), 41 (9.8)	7 – Moderate Risk
Hendrickson et al, (2022), USA [24].	39.6, n = 139 (NA)	Cross-sectional, Web-based	EMS personnel	Professional	Depression (PHQ-9), Anxiety (GAD-7)	100 (77.8), 101 (72.9)	7 – Moderate Risk
Jindal et al, (2020), India [25]	NA, n = 232 (NA)	Cross-sectional, Web-based	Paramedics	Student	Anxiety (GAD-7)	68 (29.3)	8 – Moderate Risk
Pazmino Erazo et al, (2021), Ecuador [26]	NA, n = 27 (16/11)	Cross-sectional, Web-based	Paramedics	Professional	Depression (PHQ-9), Anxiety (GAD-7)	21 (77.8), 23 (85.2)	7 – Moderate Risk
Petrie et al, (2022), Australia [27]	NA, n = 95 (NA)	Cross-sectional, Web-based	Paramedics	Professional	Depression (PHQ-9), Anxiety (GAD-7)	33 (34.7), 51 (53.7)	7 – Moderate Risk
Saeed et al, (2021), Sri Lanka, Pakistan, India [28]	NA, n = 121 (NA)	Cross-sectional, Web-based	Paramedics	Professional	Depression (SRQ-20),	43 (35.5)	7 – Moderate Risk
Sharma et al, (2021), Nepal [29]	NA, n = 25 (15/10)	Cross-sectional, Web-based	Paramedics	Professional	Depression (DASS-21), Anxiety (DASS-21), Stress (DASS-21)	5 (20.0), 7 (28.0), 4 (16.0)	7 – Moderate Risk
Skoda et al, (2020), Germany [30]	NA, n = 221 (164/55)	Cross-sectional, Web-based	Paramedics	Professional	Anxiety (GAD-7)	10 (4.5)	7 – Moderate Risk
Tsehay et al, (2021), Ethiopia [31]	34.0, n = 385 (321/64)	Cross-sectional, Self-administered	Police	Professional	Depression (PHQ-9), Anxiety (GAD-7)	111 (28.8), 116 (30.1)	9 – Low Risk
Vujanovic et al, (2021), USA [32]	47.6, n = 189 (149/40)	Cross-sectional, Self-administered	EMS personnel	Professional	Depression (ODSIS), Anxiety (OASIS)	32 (16.8), 35 (18.4)	9 – Low Risk
Williams et al, (2021), Australia [33]	NA, n = 151 (36/113)	Cross-sectional, Self-administered	Paramedics	Student	Anxiety (GAD-7)	94 (62.3)	8 – Moderate Risk
Wright et al, (2020), USA [34]	42.9, n = 473 (NA)	Cross-sectional, Web-based	EMS personnel	Professional	Depression (PHQ-8), Anxiety (GAD-7)	95 (20.1), 76 (16.1)	9 – Low Risk
Yuan et al, (2020), China [35]	36.2, n = 3517 (2960/557)	Cross-sectional, Web-based	Police	Professional	Depression (PHQ-9), Anxiety (GAD-7)	428 (20.1), 309 (16.1)	9 – Low Risk

DASS – Depression, Anxiety and Stress Scale, EMS – Emergency Medical Services, IES-R – Impact of Event Scale – Revised, GAD – Generalized Anxiety Disorder, HADS – Hospital Anxiety and Depression Scale, OASIS – Overall Anxiety Severity and Impairment Scale, ODSIS – Overall Depression Severity and Impairment Scale, PHQ – Patient Health Questionnaire, SRQ – Self-Reporting Questionnaire, USA – United States of America, NA – not applicable

For the study outcomes, 14 studies examined depression, 16 studies examined anxiety, and three studies examined stress. The prevalence of depression ranged from 11.4% to 77.8%. Depression was measured using the Depression, Anxiety and Stress Scale-21 (DASS-21), Patient Health Questionnaire-9 (PHQ-9), Patient Health Questionnaire-8 (PHQ-8), Patient Health Questionnaire-4 (PHQ-4), Patient Health Questionnaire-2 (PHQ-2), Hospital Anxiety and Depression Scale (HADS), Self-Reporting Questionnaire-20 (SRQ-20), and Overall Depression Severity and Impairment Scale (ODSIS). The prevalence of anxiety ranged from 9.8% to 85.2%. Anxiety was measured using Generalized Anxiety Disorder-7 (GAD-7), GAD-2, DASS-21, HADS, PHQ-4, and Overall Anxiety Severity and Impairment Scale (OASIS). The prevalence of stress ranged from 7.6% to 33.3%. Stress was measured using DASS-21 and Impact of Event Scale-Revised (IES-R) (**Table 1**).

### Prevalence of depression among first responders

**Figure 2:**
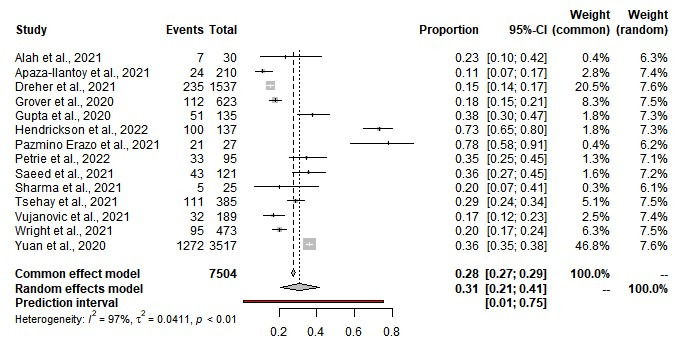
Prevalence of depression among first responders.

The pooled prevalence of depression among first responders for medical emergencies was estimated at 31% (95% CI = 21%-41%) with 67% for mild depression, 24% for moderate depression, and 16% for severe depression. We observed statistical heterogeneity among the included studies (Q = 599.64, τ^2^ = 0.0188, *I*^2^ = 97%; *P* = 0.01) (**Table 2**, **Figure 2**). The Egger regression co-efficient was 1.75 (t-value = 3.05, *P* = 0.578) and the funnel plot showed no evidence of publication bias (Figure S1 in the **Online Supplementary Document**). Among first responders, the prevalence of depression was 37% for paramedics, 28% for EMS personnel, and 22% for police. The prevalence of depression was 39% for South America, 34% for North America, 30% for Asia, 29% for Africa, and 15% for Europe.

**Table 2:** Prevalence of depression, anxiety, and stress among first responders

Outcome	n	Prevalence (95% CI)	N	*I^2^*	τ^2^	*P-*value
**Overall depression**	14	31% (21%-41%)	7504	97%	0.0411	<0.01
Mild depression	6	67% (64%-70%)	1440	42%	0.0000	0.12
Moderate depression	6	24% (17%-31%)	1440	31%	0.0027	0.21
Severe depression	4	16% (4%-34%)	1411	76%	0.0305	<0.01
**Overall anxiety**	16	32% (20%-44%)	7795	98%	0.0635	<0.01
Mild anxiety	6	60% (46%-73%)	1468	86%	0.0218	<0.01
Moderate anxiety	7	27% (14%-42%)	1475	85%	0.0320	<0.01
Severe anxiety	7	14% (7%-22%)	1475	77%	0.0097	<0.01
**Overall stress**	3	17% (4%-34%)	265	85%	0.0252	<0.01
Mild stress	2	58% (38%-77%)	26	0%	0.0000	0.34
Moderate stress	2	22% (5%-44%)	26	27%	0.0073	0.24
Severe stress	2	19% (5%-37%)	26	0%	0.0000	0.90

CI – confidence interval, n – number of studies, N – sample size

### Prevalence of anxiety among first responders

**Figure 3:**
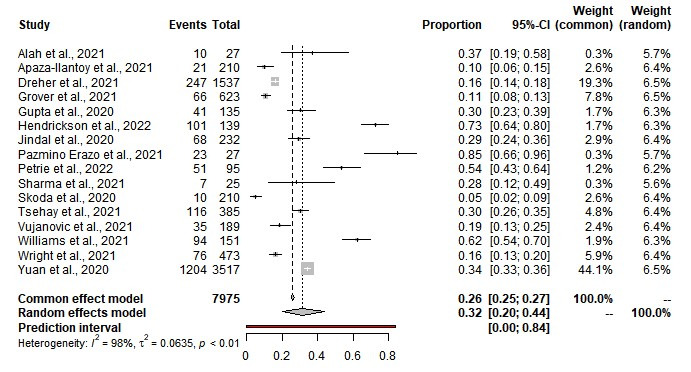
Prevalence of anxiety among first responders.

The pooled prevalence of anxiety among first responders for medical emergencies was estimated at 32% (95% CI = 20%-44%); 60% for mild anxiety, 27% for moderate anxiety, and 14% for severe anxiety. We observed statistical heterogeneity among the included studies (Q = 975.41, τ^2^ = 0.0323, *I^2^* = 98%; *P* = 0.01) (**Table 2**, **Figure 3**). The Egger regression co-efficient was 4.02 (t-value = 3.40, *P* = 0.257) and the funnel plot showed no evidence of publication bias (Figure S1 in the **Online Supplementary Document**). Among first responders, the prevalence of anxiety was 38% for paramedics, 28% for EMS personnel, and 19% for police. The prevalence of anxiety was 44% for South America, 34% for Asia, 33% for North America, 30% for Africa, and 9% for Europe.

### Prevalence of stress among first responders

**Figure 4:**
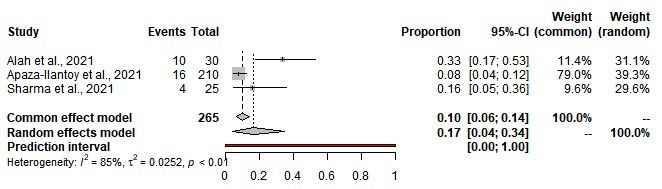
Prevalence of stress among first responders.

The pooled prevalence of stress among first responders for medical emergencies was estimated at 17% (95% CI = 4%-36%); 58% for mild stress, 22% for moderate stress, and 19% for severe stress. We observed statistical heterogeneity among the included studies (τ^2^ = 0.0295, *I^2^* = 85%; *P* = 0.01) (**Table 2**, **Figure 4**).

### Associated factors for depression and anxiety

The study findings showed that marital status (married and unmarried) was significantly associated with depression, while gender and being a first responder (including paramedics and EMS personnel) were not. Married first responders (OR = 1.50, 95% CI = 1.26-1.78) were more likely to be depressed, while unmarried first responders (OR = 0.67, 95% CI = 0.56-0.79) were less likely. First responders (OR = 1.25, 95% CI = 0.92-1.70) were non-significantly associated with being depressed compared to other health care workers. Among first responders, paramedics (OR = 1.34, 95% CI = 0.88-2.04) and EMS personnel (OR = 1.18, 95% CI = 0.72-1.93) were non-significantly associated with being depressed. Considering gender, male first responders with (OR = 0.87, 95% CI = 0.72-1.04) were non-significantly associated with a reduced risk of being depressed while female first responders with (OR = 1.15, 95% CI = 0.96-1.38) were non-significantly associated with increased risk of being depressed.

Similarly, the study findings showed that marital status (married and unmarried) was significantly associated with anxiety while gender and being a first responder (including paramedics and EMS personnel) were not. Married first responders (OR = 1.94, 95% CI = 1.62-2.33) were more likely to be anxious, while unmarried first responders (OR = 0.52, 95% CI = 0.43-0.63) were less likely. First responders (OR = 1.03, 95% CI = 0.71-1.49) were non-significantly associated with being anxious compared to other health care workers. Among first responders, paramedics (OR = 1.00, 95% CI = 0.75-1.34) were non-significantly associated with being anxious, while EMS personnel (OR = 0.83, 95% CI = 0.53-1.28) were non-significantly associated with reduced risk of being anxious. Male first responders with (OR = 0.73, 95% CI = 0.41-1.30) were non-significantly associated with a reduced risk of being anxious while female first responders (OR = 1.32, 95% CI = 0.82-2.13) were non-significantly associated with increased risk of being anxious (**Table 3**).

**Table 3:** Associated factors for depression and anxiety among first responders

		Depression		Anxiety		
**Characteristics**	**n**	**OR (95% CI)**	***P-*value**	**n**	**OR (95% CI)**	***P-*value**
**First responder**	8	1.21 (0.90-1.62)	0.200	8	1.01 (0.72-1.42)	0.935
Paramedics	6	1.34 (0.88-2.04)	0.175	6	1.18 (0.72-1.93)	0.516
EMS personnel	2	1.00 (0.75-1.34)	0.977	2	0.83 (0.53-1.28)	0.389
**Gender**						
Male	2	0.87 (0.72-1.04)	0.129	3	0.73 (0.41-1.30)	0.288
Female	2	1.15 (0.96-1.38)	0.129	3	1.32 (0.82-2.13)	0.257
**Marital** **status**						
Married	2	1.50 (1.26-1.78)	**0.000**	2	1.94 (1.62-2.33)	**0.000**
Unmarried	2	0.67 (0.56-0.79)	**0.000**	2	0.52 (0.43-0.63)	**0.000**

CI – confidence interval, EMS – emergency medical services, n – number of studies, OR – odds ratio

### Results of the sub-group analysis for depression and anxiety

The results of subgroup analysis for the prevalence of depression demonstrated that type of interview, study quality, sample size, and study type were significant moderator variables while country status, setting, and assessment tool were not. Regarding the type of interview (*P* = 0.0007), structured interviews had a prevalence of 33% compared to a prevalence of 16% for unstructured interviews. Study quality showed to be a significant moderator (*P* = 0.0005), as moderate quality studies had a prevalence of 43% compared to a prevalence of 20% for high-quality studies. Regarding sample size (*P* = 0.006), studies with sample sizes <200 had a prevalence of 39% compared to a prevalence of 21% for studies with sample sizes ≥200. Regarding study type (*P* = 0.069), web-based studies had a prevalence of 34% compared to a prevalence of 18% for interview-based studies. The country status prevalence rates (*P* = 0.825) were 28% for high income, 31% for middle income, and 29% for low-income countries. The prevalence rates by setting (*P* = 0.719) were 31% for urban and rural and 27% for urban settings. Regarding assessment tools (*P* = 0.200), studies that used Patient Health Questionnaire had a prevalence of 34% compared to a prevalence of 23% for studies that used other types of assessment tools. Concerning the number of cases and mortality rate (*P* = 0.859), top-5 countries had a prevalence of 31% compared to a prevalence of 29% for non-top-5 countries, respectively.

The results of sub-group analysis for the prevalence of anxiety revealed that type of interview, study quality, and sample size were significant moderator variables while study type, country status, setting, and assessment tool were not. Based on the interview type (*P* = 0.0012), structured interviews had a prevalence of 33% compared to a prevalence of 13% for unstructured interviews. Study quality showed to be a significant moderator (*P* = 0.014), moderate quality studies had a prevalence of 42% compared to a prevalence of 18% for high-quality studies. Regarding sample size (*P* = 0.0005), studies with sample sizes <200 had a prevalence of 48% compared to a prevalence of 17% for studies with sample sizes ≥200. Regarding study type (*P* = 0.293), web-based studies had a prevalence of 26% compared to a prevalence of 44% for face-to-face studies. The country status prevalence rates (*P* = 0.921) were 30% for high-income and 29% for middle-income and low-income countries. The prevalence rates by setting (*P* = 0.847) were 29% for urban and rural and 31% for urban. For the assessment tool (*P* = 0.228), studies that used GAD questionnaire had a prevalence of 31% compared to a prevalence of 22% for studies that used other types of assessment tools. Accounting for the number of cases and mortality rate (*P* = 0.675), top-5 countries had a prevalence of 27% compared to a prevalence of 32% for non-top-5 countries, respectively (Table S3 in the **Online Supplementary Document**).

## DISCUSSION

### Prevalence of depression, anxiety, and stress

To our knowledge, this is the first meta-analysis exploring the pooled prevalence of depression, anxiety, and stress among first responders for medical emergencies during the COVID-19 pandemic. We found that the pooled prevalence was 31% for depression, 32% for anxiety, and 17% for stress. There is a high prevalence of mild depression, anxiety, and stress was high, followed by moderate and severe types. The current study findings show an increase in the prevalence of depression, anxiety, and stress compared to the pre-COVID-19 pandemic period [6]. A previous meta-analysis study by Petrie et al. [9] found that the pooled prevalence of depression and anxiety was 15% among ambulance personnel. Furthermore, a recent meta-analysis study by Fan, Gao & Zhang [8] revealed that the prevalence of depression among health care workers during the SARS and MERS epidemics was 19.4%. The possible explanation for the observed higher rates of depression, anxiety, and stress among first responders during the COVID-19 pandemic could be that the COVID-19 pandemic has had a larger impact globally than the MERS and SARS epidemics, including the number of infected patients and death [8]. Moreover, first responders may have encountered an increased number of patients infected with or deceased from COVID-19, as they work in uncontrolled environments (including patient’s home, office, on the streets). They might have also been affected by limited protective resources and increased workload during the COVID-19 pandemic. Therefore, the provision of work resources (including protective equipment) to ensure and improve the safety of first responders might eventually lead to improved psychological well-being and better delivery of pre-hospital medical services.

Among first responders, paramedics and EMS personnel demonstrated a higher prevalence of depression and anxiety compared to police personnel. The possible explanation could be the nature of the work of these first responders when dealing with COVID-19 patients. Paramedics and EMS personnel are more likely to have direct contact with COVID-19 patients and witness the suffering and even death of COVID-19 patients compared to the police. As such, assessment of depression and anxiety among paramedics and EMS personnel should be encouraged to ensure prompt psychotherapeutic interventions are delivered to prevent short-term and long-term consequences. Regarding marital status, married responders were more likely at risk of depression and anxiety compared to unmarried ones. Married responders may have too many family-related responsibilities and may worry about their significant others contracting COVID-19, thereby possibly having a higher risk for depression and anxiety compared to unmarried responders. Therefore, early assessment and prompt management of mild depression, anxiety, and stress among first responders to prevent progress into moderate and severe types and associated short-term and long-term consequences should be encouraged, as well as maintaining the general health and psychological well-being of these front-line workers to ensure good delivery of pre-hospital medical services. However, due to limited information included in this study, we were unable to provide more individual and work-related factors besides being first responder, gender, and marital status, which could help in a comprehensive understanding of the nature of depression, anxiety, and stress. Future studies should explore the association of more individual-related and work-related factors with depression, anxiety, and stress among first responders.

### Moderator variables for depression and anxiety

The sub-group analysis revealed that type of interview, study quality, sample size, and study type were significant moderator variables for the prevalence of depression and anxiety. Regarding the type of interview, studies that used unstructured assessment tools showed to have a lower prevalence of depression and anxiety compared to studies that used structured assessment tools. Structured assessment tools may have a comprehensive view and constructs to measure depression and anxiety compared to unstructured assessment tools, which may have limited information. The use of unstructured assessment tools in the measurement of depression and anxiety may likely underestimate the prevalence of depression and anxiety. Future studies using such tools should avoid the underestimation of the prevalence rate. Moderate quality studies demonstrated to have a higher prevalence of depression and anxiety compared to high-quality studies. Moderate quality studies may have more bias in the internal and external validity, which may likely contribute to the overestimation of the prevalence of depression and anxiety. Studies with a sample size <200 were revealed to have a higher prevalence of depression and anxiety compared to studies with a sample size ≥200. Studies with sample size <200 were more likely to be underpowered and thus overestimate the prevalence of depression and anxiety. To ensure a higher quality, future studies should use larger sample sizes in assessing the prevalence of depression and anxiety among first responders.

### Strengths and limitations

Our meta-analysis has several strengths. First, it is the first to provide comprehensive evidence on the prevalence of depression, anxiety, and stress among first responders during the COVID-19 pandemic. Second, we performed a comprehensive search to identify potentially eligible studies and the inclusion criteria did not have any language limitations. Third, we followed the MOOSE guidelines in the conduct and reporting of the current meta-analysis and registered the study protocol with PROSPERO for better research integrity and transparency. However, there are some study limitations to be considered. First, statistical heterogeneity was observed in all the outcomes and the moderator analysis was used to explore the potential moderator variables to explain the source of the heterogeneity. Second, due to limited information, we were unable to explore and examine the influence of individual-related factors (age, education, smoking and alcohol status, and coping mechanism), and work-related factors (years of work experience, prior training, type of work, peer support, communication, lack of rest, near death experience, severity of causalities, previous exposure to disaster, contact with corpses, and awareness of support measures).

## CONCLUSIONS

The current meta-analysis shows a substantial higher prevalence of depression, anxiety, and stress among first responders during the COVID-19 pandemic. Early assessment and management of mild depression, anxiety, and stress should be encouraged to prevent the development of moderate and severe types in order to ensure psychological well-being among first responders. Essential support initiatives and interventions for the management of depression, anxiety, and stress among first responders should be developed to prevent short-term and long-term consequences of these negative psychological outcomes. Future high-quality studies with larger sample sizes reporting individual-related and work-related factors should be encouraged to provide a comprehensive view into the nature of depression, anxiety, and stress among first responders.

## Additional material


Online Supplementary Document
