# Supplementary material for: Prevalence of depression, anxiety, and stress among first responders for medical emergencies during COVID-19 pandemic: A meta-analysis
Source: J Glob Health. 2022 Jul 25;12:05028. doi: 10.7189/jogh.12.05028 (PMC9309001; doi:10.7189/jogh.12.05028)

Prevalence of Depression, Anxiety, and Stress among First Responders for Medical Emergencies during COVID-19 Pandemic: A Meta-Analysis

Online Supplementary Document

Table S1: Search Strategy……………………….…………………….…………………….…2

Table S2: Risk of Bias Assessment……………………………….…………….……..…….…6

Table S3: Moderator Analysis…………...………………………….………….…...…….7

Figure S1: Funnel Plot for Publication Bias for Depression……............................….………..9

Figure S2: Funnel Plot for Publication Bias for Anxiety……...………………..…………..…10

Table S1: Search Strategy

Database	Mesh-Terms	Number of studies
CINAHL	"TX (prevalence OR incidence OR epidemiology OR rate OR rates OR number OR proportion OR probability OR event) AND TX (depression OR anxiety OR stress OR psychological distress) AND TX (Emergency Medical Services Personnel OR EMS personnel OR ambulance personnel OR fire fighters OR police OR first responders OR paramedics OR emergency medical technicians OR EMTs) AND TX (COVID-19 or covid-19 OR Corona virus OR SARS-COV 2)	138
Embase	(prevalence:ab,ti OR incidence:ab,ti OR epidemiology:ab,ti OR rate:ab,ti OR rates:ab,ti OR number:ab,ti OR proportion:ab,ti OR probability:ab,ti OR event:ab,ti) AND (depression:ab,ti OR anxiety:ab,ti OR stress:ab,ti OR 'psychological distress':ab,ti) AND ('emergency medical services personnel':ab,ti OR 'ems personnel':ab,ti OR 'ambulance personnel':ab,ti OR 'fire fighters':ab,ti OR police:ab,ti OR 'first responders':ab,ti OR paramedics:ab,ti OR 'emergency medical technicians':ab,ti OR emts:ab,ti) AND ('covid 19':ab,ti OR 'corona virus':ab,ti OR 'sars-cov 2':ab,ti)	42
PubMed	("epidemiology"[MeSH Subheading] OR "epidemiology"[All Fields] OR "prevalence"[All Fields] OR "prevalence"[MeSH Terms] OR "prevalance"[All Fields] OR "prevalences"[All Fields] OR "prevalence s"[All Fields] OR "prevalent"[All Fields] OR "prevalently"[All Fields] OR "prevalents"[All Fields] OR ("epidemiology"[MeSH Subheading] OR "epidemiology"[All Fields] OR "incidence"[All Fields] OR "incidence"[MeSH Terms] OR "incidences"[All Fields] OR "incident"[All Fields] OR "incidents"[All Fields]) OR ("epidemiologies"[All Fields] OR "epidemiology"[MeSH Subheading] OR "epidemiology"[All Fields] OR "epidemiology"[MeSH Terms] OR "epidemiology s"[All Fields]) OR ("j rehabil assist technol eng"[Journal] OR "rate"[All Fields]) OR "rates"[All Fields] OR ("number"[All Fields] OR "numbers"[All Fields]) OR ("proportion"[All Fields] OR "proportions"[All Fields]) OR ("probability"[MeSH Terms] OR "probability"[All Fields] OR "probabilities"[All Fields]) OR ("event"[All Fields] OR "event s"[All Fields] OR "events"[All Fields])) AND ("depressed"[All Fields] OR "depression"[MeSH Terms] OR "depression"[All Fields] OR "depressions"[All Fields] OR "depression s"[All Fields] OR "depressive disorder"[MeSH Terms] OR ("depressive"[All Fields] AND "disorder"[All Fields]) OR "depressive disorder"[All Fields] OR "depressivity"[All Fields] OR "depressive"[All Fields] OR "depressively"[All Fields] OR	258

	"depressiveness"[All Fields] OR "depressives"[All Fields] OR ("anxiety"[MeSH Terms] OR "anxiety"[All Fields] OR "anxieties"[All Fields] OR "anxiety s"[All Fields]) OR ("stress"[All Fields] OR "stressed"[All Fields] OR "stresses"[All Fields] OR "stressful"[All Fields] OR "stressfulness"[All Fields] OR "stressing"[All Fields]) OR ("psychological distress"[MeSH Terms] OR ("psychological"[All Fields] AND "distress"[All Fields]) OR "psychological distress"[All Fields])) AND ((("emergency medical services"[MeSH Terms] OR ("emergency"[All Fields] AND "medical"[All Fields] AND "services"[All Fields]) OR "emergency medical services"[All Fields]) AND ("occupational groups"[MeSH Terms] OR ("occupational"[All Fields] AND "groups"[All Fields]) OR "occupational groups"[All Fields] OR "personnel"[All Fields] OR "personnel s"[All Fields] OR "personnels"[All Fields])) OR (("emerg med serv"[Journal] OR "ems mag"[Journal] OR "ems"[All Fields]) AND ("occupational groups"[MeSH Terms] OR ("occupational"[All Fields] AND "groups"[All Fields]) OR "occupational groups"[All Fields] OR "personnel"[All Fields] OR "personnel s"[All Fields] OR "personnels"[All Fields])) OR (("ambulance s"[All Fields] OR "ambulances"[MeSH Terms] OR "ambulances"[All Fields] OR "ambulance"[All Fields]) AND ("occupational groups"[MeSH Terms] OR ("occupational"[All Fields] AND "groups"[All Fields]) OR "occupational groups"[All Fields] OR "personnel"[All Fields] OR "personnel s"[All Fields] OR "personnels"[All Fields])) OR ("firefighters"[MeSH Terms] OR "firefighters"[All Fields] OR ("fire"[All Fields] AND "fighters"[All Fields]) OR "fire fighters"[All Fields]) OR ("police"[MeSH Terms] OR "police"[All Fields] OR "polices"[All Fields] OR "police s"[All Fields] OR "policed"[All Fields] OR "policing"[All Fields]) OR ("emergency responders"[MeSH Terms] OR ("emergency"[All Fields] AND "responders"[All Fields]) OR "emergency responders"[All Fields] OR ("first"[All Fields] AND "responders"[All Fields]) OR "first responders"[All Fields]) OR ("allied health personnel"[MeSH Terms] OR ("allied"[All Fields] AND "health"[All Fields] AND "personnel"[All Fields]) OR "allied health personnel"[All Fields] OR "paramedics"[All Fields] OR "emergency medical technicians"[MeSH Terms] OR ("emergency"[All Fields] AND "medical"[All Fields] AND "technicians"[All Fields]) OR "emergency medical technicians"[All Fields] OR "paramedic"[All Fields] OR "paramedic s"[All Fields] OR "paramedical"[All Fields] OR "paramedicals"[All Fields]) OR ("emergency medical technicians"[MeSH Terms] OR ("emergency"[All Fields] AND	

	"medical"[All Fields] AND "technicians"[All Fields]) OR "emergency medical technicians"[All Fields]) OR "EMTs"[All Fields]) AND ("covid 19"[All Fields] OR "covid 19"[MeSH Terms] OR "covid 19 vaccines"[All Fields] OR "covid 19 vaccines"[MeSH Terms] OR "covid 19 serotherapy"[All Fields] OR "covid 19 serotherapy"[Supplementary Concept] OR "covid 19 nucleic acid testing"[All Fields] OR "covid 19 nucleic acid testing"[MeSH Terms] OR "covid 19 serological testing"[All Fields] OR "covid 19 serological testing"[MeSH Terms] OR "covid 19 testing"[All Fields] OR "covid 19 testing"[MeSH Terms] OR "sars cov 2"[All Fields] OR "sars cov 2"[MeSH Terms] OR "severe acute respiratory syndrome coronavirus 2"[All Fields] OR "ncov"[All Fields] OR "2019 ncov"[All Fields] OR (("coronavirus"[MeSH Terms] OR "coronavirus"[All Fields] OR "cov"[All Fields]) AND 2019/11/01:3000/12/31[Date - Publication]) OR ("covid 19"[All Fields] OR "covid 19"[MeSH Terms] OR "covid 19 vaccines"[All Fields] OR "covid 19 vaccines"[MeSH Terms] OR "covid 19 serotherapy"[All Fields] OR "covid 19 serotherapy"[Supplementary Concept] OR "covid 19 nucleic acid testing"[All Fields] OR "covid 19 nucleic acid testing"[MeSH Terms] OR "covid 19 serological testing"[All Fields] OR "covid 19 serological testing"[MeSH Terms] OR "covid 19 testing"[All Fields] OR "covid 19 testing"[MeSH Terms] OR "sars cov 2"[All Fields] OR "sars cov 2"[MeSH Terms] OR "severe acute respiratory syndrome coronavirus 2"[All Fields] OR "ncov"[All Fields] OR "2019 ncov"[All Fields] OR (("coronavirus"[MeSH Terms] OR "coronavirus"[All Fields] OR "cov"[All Fields]) AND 2019/11/01:3000/12/31[Date - Publication])) OR (("corona"[All Fields] OR "coronae"[All Fields] OR "coronas"[All Fields]) AND ("virology"[MeSH Subheading] OR "virology"[All Fields] OR "viruses"[All Fields] OR "viruses"[MeSH Terms] OR "virus s"[All Fields] OR "viruse"[All Fields] OR "virus"[All Fields])) OR ("sars cov 2"[MeSH Terms] OR "sars cov 2"[All Fields] OR "sars cov 2"[All Fields]))	
Psych Info	TX (prevalence OR incidence OR epidemiology OR rate OR rates OR number OR proportion OR probability OR event) AND TX (depression OR anxiety OR stress OR psychological distress) AND TX (Emergency Medical Services Personnel OR EMS personnel OR ambulance personnel OR fire fighters OR police OR first responders OR paramedics OR emergency medical technicians OR EMTs) AND TX (COVID-19 or covid-19 OR Corona virus OR SARS-COV 2)	42

Web of Science	prevalence OR incidence OR epidemiology OR rate OR rates OR number OR proportion OR probability OR event **(All Fields) and** depression OR anxiety OR stress OR psychological distress **(All Fields) and** Emergency Medical Services Personnel OR EMS personnel OR ambulance personnel OR fire fighters OR police OR first responders OR paramedics OR emergency medical technicians OR eats **(All Fields) and** COVID-19 or covid-19 OR Corona virus OR SARS-COV 2 **(All Fields)**<br>Timespan: All years. Indexes: SCI-EXPANDED, SSCI.	250
WHO COVID-19 Database	(prevalence OR incidence OR epidemiology OR rate OR rates OR number OR proportion OR probability OR event) AND (depression OR anxiety OR stress OR psychological distress) AND (Emergency Medical Services Personnel OR EMS personnel OR ambulance personnel OR fire fighters OR police OR first responders OR paramedics OR emergency medical technicians OR EMTs) AND (COVID-19 or covid-19 OR Corona virus OR SARS-COV 2)	33

Table S2: Risk of Bias Assessment

Study	External validity				Internal validity						Total
	Representativeness	Sampling frame	Random selection	Non-response	Data collection	Case definition	Instrument	Same mode	Prevalence period	Numerator / denominator	
Alah, 2021	0	0	0	1	1	1	1	1	1	1	7 – M
Apaza-Llantoy, 2021	1	1	0	1	1	1	1	1	1	9	9 – L
Dreher, 2021	1	1	0	1	1	1	1	1	1	1	9 – L
Grover, 2020	1	1	0	1	1	1	1	1	1	1	9 – L
Gupta, 2020	0	0	0	1	1	1	1	1	1	1	7 – M
Hendrickson, 2020	0	0	0	1	1	1	1	1	1	1	7 – M
Jindal, 2020	1	0	0	1	1	1	1	1	1	1	8 – M
Pazmino Erazo, 2020	0	0	0	1	1	1	1	1	1	1	7 – M
Petrie, 2022	0	0	0	1	1	1	1	1	1	1	7 – M
Saeed, 2022	0	0	0	1	1	1	1	1	1	1	7 – M
Sharma, 2021	0	0	0	1	1	1	1	1	1	1	7 – M
Skoda, 2021	0	0	0	1	1	1	1	1	1	1	7 – M
tsehay, 2021	1	1	0	1	1	1	1	1	1	1	9 – L
Vujanovic, 2021	1	1	0	1	1	1	1	1	1	1	9– L
Williams, 2021	1	1	0	0	1	1	1	1	1	1	8 – M
Wright, 2021	1	1	0	1	1	1	1	1	1	1	9 – L
Yuan, 2020	1	1	0	1	1	1	1	1	1	1	9 – L

Score of 1 for low risk and 0 for high risk. Poor quality: score of ≤ 6 (H), moderate quality: score of 7 or 8 (M), high quality: score of 9 or 10 (L)

Table S3: Sub-group Analysis

Depression				Anxiety			
Characteristics	*P-value*	n	Prevalence (95%CI)	Characteristics	*P-value*	n	Prevalence (95%CI)
First responders	0·224	14		First responders	0·254	16	
Paramedics		6	37% (25% – 52%)	Paramedics		8	38% (20% – 60%)
EMS personnel		4	28% (12% – 54%)	EMS personnel		4	28% (11% – 53%)
Police		4	22% (13% – 33%)	Police		4	19% (10% – 32%)
Continent	**<0·0001**	14		Continent	**0·057**	16	
South America		3	39% (6% – 87%)	South America		2	44% (4% – 93%)
North America		2	34% (12% – 66%)	Asia		8	34% (23% – 47%)
Asia		7	30% (23% – 37%)	North America		3	33% (11% – 65%)
Africa		1	29% (25% – 34%)	Africa		1	30% (26% – 35%)
Europe		1	15% (14% – 17%)	Europe		2	9% (4% – 21%)
Type of interview	**0·0007**	14		Type of interview	**0·0012**	16	
Structured		12	33% (22% – 45%)	Structured		14	33% (21% – 48%)
Unstructured		2	16% (15% – 18%)	Unstructured		2	13% (10% – 18%)
Study quality	**0·0005**	14		Study quality	**0·014**	16	
Moderate risk		7	43% (28% – 60%)	Moderate risk		9	42% (24% – 63%)
Low risk		7	20% (15% – 26%)	Low risk		7	18% (13% – 25%)
Sample size	**0·028**	14		Sample size	**0·0005**	16	
<200		8	39% (24% – 55%)	<200		8	48% (32% – 65%)
>200		6	21% (15% – 28%)	>200		8	17% (11% – 25%)
Study design	**<0·0001**	14		Study design	**<0·0001**	16	
Prospective cohort		1	73% (65% – 80%)	Prospective cohort		1	73% (65% – 79%)
Cross-sectional		13	27% (19% – 33%)	Cross-sectional		15	27% (18% – 39%)
Study type	**0·024**	14		Study type	0·293	16	
Web-based		11	34% (23% – 47%)	Web-based		12	26% (16% – 38%)
Face-to-face		3	18% (11% – 27%)	Face-to-face		4	44% (16% – 77%)
Number of cases	0·859	14		Number of cases	0·675	16	
Top-5 countries		5	31% (16% – 51%)	Top 5 countries		6	27% (14% – 45%)
Non top-5 countries		9	29% (19% – 41%)	Non top 5 countries		10	32% (17% – 50%)
Mortality rate	0·859	14		Mortality rate	0·675	16	
Top-5 countries		5	31% (16% – 51%)	Top 5 countries		6	27% (20% – 45%)

Non top-5 countries		9	29% (19% – 41%)	Non top 5 countries		10	32% (17% – 50%)
Country status	0·825	14		Country status	0·921	16	
High-income		6	28% (16% – 46%)	High income		8	30% (15% – 51%)
Middle and Low-income		8	31% (20% – 44%)	Middle and Low-income		8	29% (16% – 46%)
Setting		14		Setting	0·847	16	
Urban & rural	0·719	9	31% (21% – 43%)	Urban & rural		9	29% (15% – 48%)
Urban		5	27% (13% – 48%)	Urban		7	31% (17% – 50%)
Assessment tool	0·200	14		Assessment tool	0·228	16	
PHQ		9	34% (21% – 49%)	GAD		10	35% (19% – 54%)
Other		5	23% (14% – 34%)	Other		6	22% (13% – 36%)

CI: confidence interval, GAD: Generalized Anxiety Disorder, n: number of studies, p-value: probability value, PHQ: Patient Health Questionnaire

Figure S1: Funnel Plot for Publication Bias for Depression

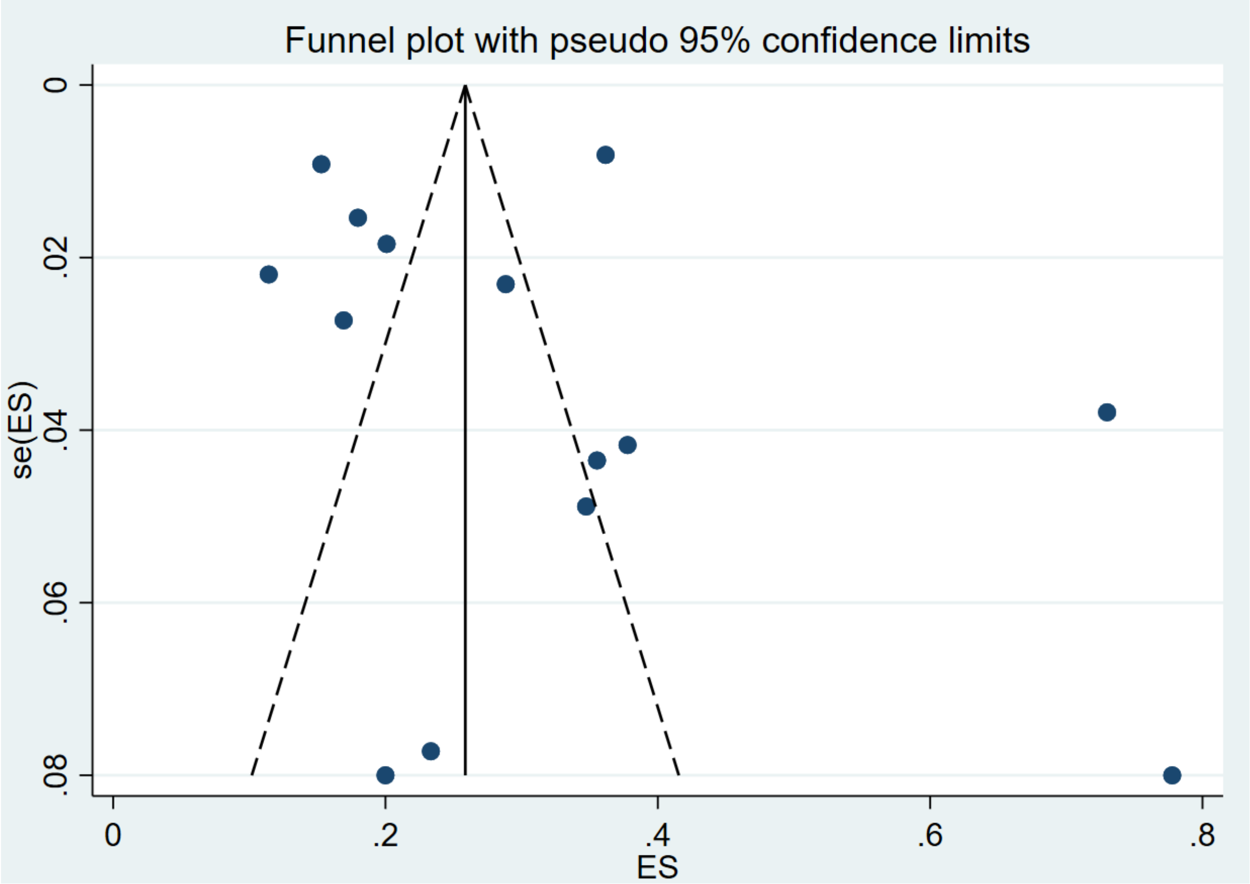

Figure S2: Funnel Plot for Publication Bias for Anxiety

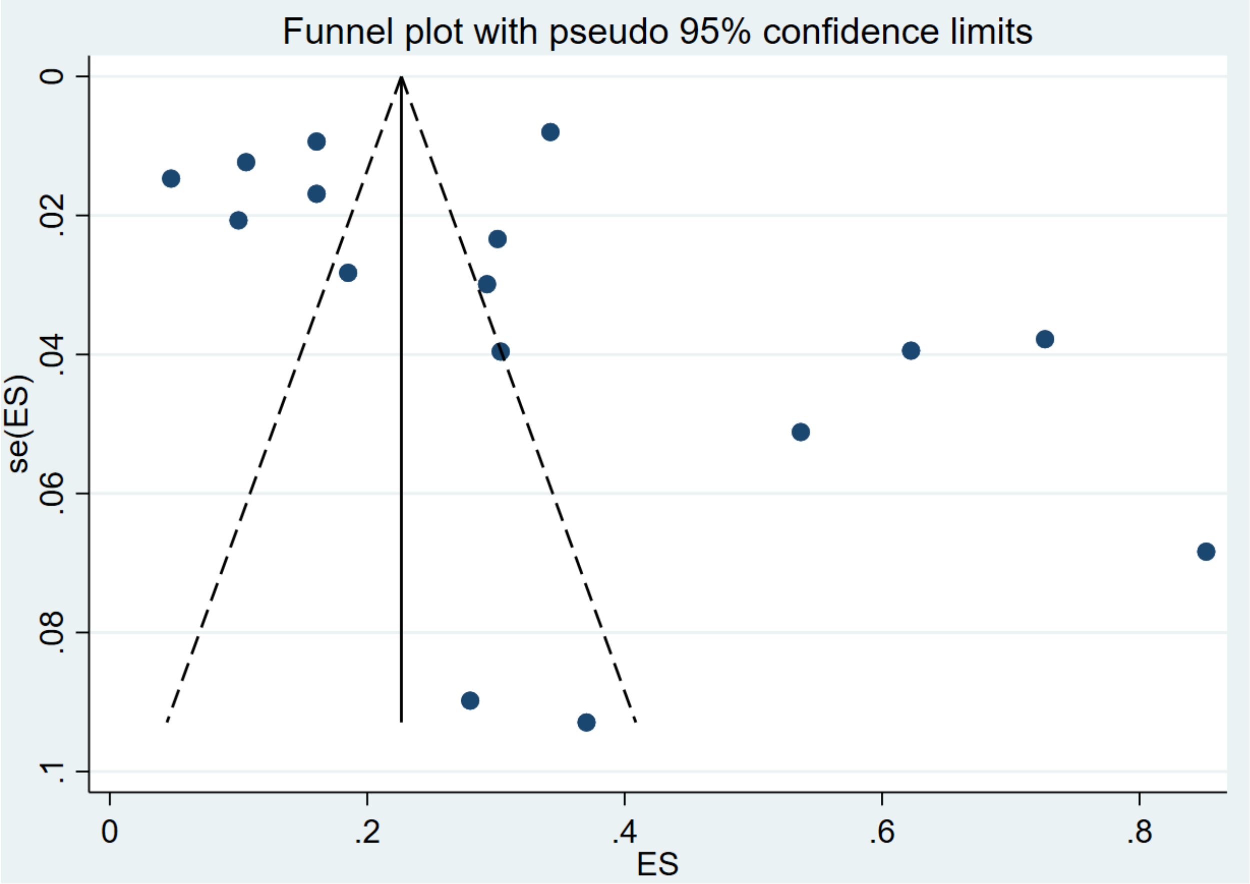

Supplement: Online Supplementary Document [file jogh-12-05028-s001.pdf]
